# Supplementary material for: Acinetobacter nosocomialis Causes as Severe Disease as Acinetobacter baumannii in Northeast Thailand: Underestimated Role of A. nosocomialis in Infection
Source: Microbiol Spectr. 2022 Oct 13;10(6):e02836-22. doi: 10.1128/spectrum.02836-22 (PMC9769887; doi:10.1128/spectrum.02836-22)
Supplement: Supplemental file 1 — Tables S1 and S2 and Fig. S1 to S5. Download spectrum.02836-22-s0001.pdf, PDF file, 0.9 MB [file spectrum.02836-22-s0001.pdf]

Supplementary Table S1: Primers used in the study.

DNA region	Primer name	Primer sequence (5’ → 3’)	Reference
rpoB	rpoB-F	GGTCCTGGTGGTTTAACACG	Sherief EL et al.,
	rpoB-R	CGAATAACGATACGAGAAGCA	2015
gyrB	Sp4F	CACGCCGTAAGAGTGCATTA	Higgins et al.,
	Sp4R	AACGGAGCTTGTCAGGGTTA	2007
16s rRNA	P-Ab-ITSF	CATTATCACGGTAATAGTG	Chen et al., 2007
	P-Ab-ITSB	AGAGCACTGTGCACTTAAG	
recA	P-rA1	CCTGAATCTTCTGGTAAAAC	Krawczyk et al.,
	P-rA2	GTTTCTGGGCTGCCAAACATTAC	2002

Supplementary Table S2: Description of bacterial isolates used in the study.

Isolate No.	Multiplex PCR	specimen	ICU	Survivability	Final diagnosis	MDR
AB001	*A. baumannii*	Pus	No	Survival	Chronic wound infection	Non-MDR
AB004	*A. baumannii*	Sputum	No	Survival	Pneumonia	MDR
AB005	*A. baumannii*	Sputum	Yes	Survival	Pneumonia	Non-MDR
AB011*	*A. baumannii*	Sputum	No	Non-Survival	Pneumonia with sepsis	MDR
AB012*	*A. baumannii*	Sputum	Yes	Survival	Pneumonia with sepsis	MDR
AB015	*A. baumannii*	Pus	No	Survival	Osteomyelitis	MDR
AB021	*A. baumannii*	Sputum	No	Non-Survival	Pneumonia	MDR
AB028	*A. baumannii*	Sputum	No	Survival	Pneumonia	Non-MDR
AB035*	*A. baumannii*	Sputum	No	Survival	Pneumonia with sepsis	MDR
AN006	*A. nosocomialis*	Sputum	Yes	Survival	Pneumonia	MDR
AN023	*A. nosocomialis*	Sputum	No	Non-Survival	Pneumonia with sepsis	MDR
AN026	*A. nosocomialis*	Sputum	No	Survival	Pneumonia with sepsis	Non-MDR

* isolates used in western blot analysis

A

Sample ID	Multiplex PCR	16s Ribosomal DNA sequencing blasted with NCBI
20-001	*Acinetobacter baumannii*	*Acinetobacter baumannii*
20-002	*Acinetobacter baumannii*	*Acinetobacter baumannii*
20-004	*Acinetobacter baumannii*	*Acinetobacter baumannii*
20-005	*Acinetobacter baumannii*	*Acinetobacter baumannii*
20-006	*Acinetobacter nosocomialis*	*Acinetobacter nosocomialis*
20-008	Unidentified	*Staphylococcus sp. BRM9*
20-009	Unidentified	*Unidentified Acinetobacter sp.*
20-011	*Acinetobacter baumannii*	*Acinetobacter baumannii*
20-012	*Acinetobacter baumannii*	*Acinetobacter baumannii*
20-015	*Acinetobacter baumannii*	*Acinetobacter baumannii*
20-021	*Acinetobacter baumannii*	*Acinetobacter baumannii*
20-023	*Acinetobacter nosocomialis*	*Acinetobacter nosocomialis*
20-026	*Acinetobacter nosocomialis*	*Acinetobacter nosocomialis*
20-028	*Acinetobacter baumannii*	*Acinetobacter baumannii*
20-035	*Acinetobacter baumannii*	*Acinetobacter baumannii*
20-036	*Acinetobacter baumannii*	*Acinetobacter baumannii*
20-044	Unidentified	*Burkholderia lata strain 383*
20-046	*Acinetobacter nosocomialis*	*Acinetobacter nosocomialis*

B

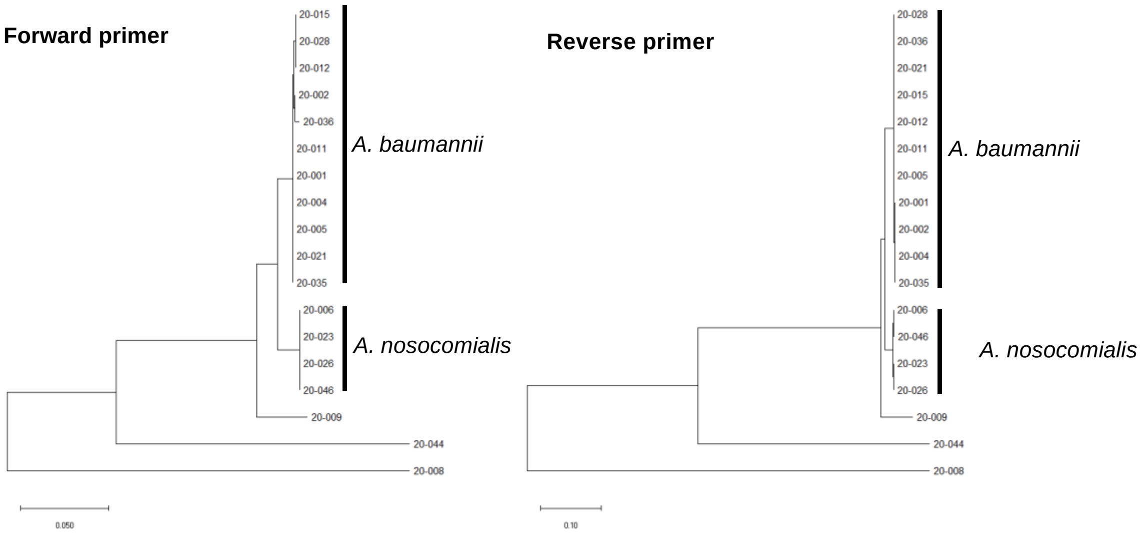

Supplementary Figure S1: Confirmation of multiplex PCR results by 16S ribosomal RNA sequencing. Multiplex PCR results of each bacterial isolates were compared with sequencing data blasted with NCBI database **(A)**. The 16S ribosomal RNA sequences from forward primer or reverse primer of each bacterial isolates were analyzed and shown as phylogenic tree **(B)**.

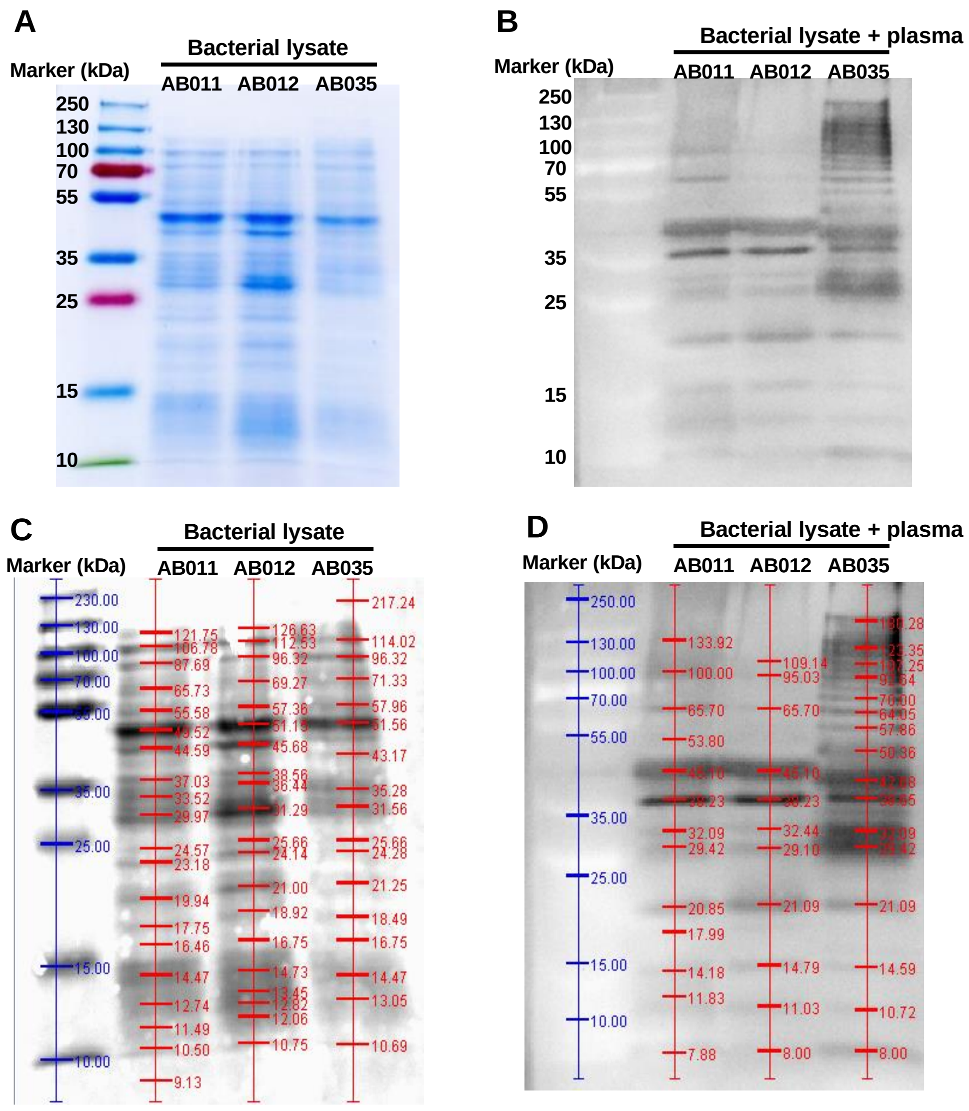

Supplementary Figure S2: Bacterial lysate separation by gel electrophoresis and human plasma IgG binding to bacterial lysate visualized by Western blotting. Bacterial lysates of clinical *A. baumannii* (500 µg) were separated by gel electrophoresis prior stained with 0.1% Coomassie blue **(A)**. Separated bacterial lysates were blotted onto PVDF membrane before probed with plasma sample. Human IgG binding on lysate proteins were tested and detected signals by using a ChemiDoc XssRS imaging system (Biorad) and analyzed with Quantity One (Biorad) software **(B)**. Both lysate protein and Western blot membrane with human IgG signals were analyzed for molecular weight of detected bands **(C and D)**.

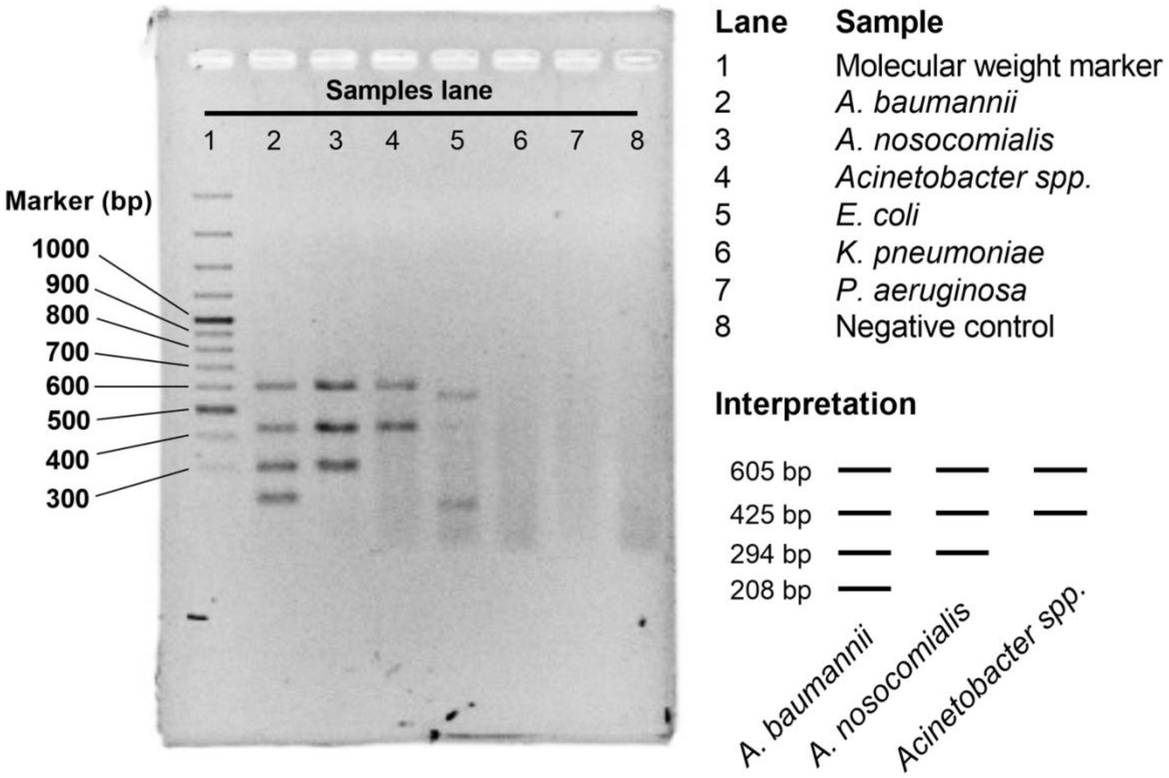

Supplementary Figure S3: Representative gel electrophoresis of multiplex PCR product from bacterial DNA and interpretation for *A. baumannii*, *A. nosocomialis*, *Acinetobacter species* or other bacteria. Multiplex PCR was performed using heat extracted bacterial DNA samples and amplicon separated on an agarose gel. DNA samples were loaded in each lane as indicated.

Plasma samples (IgG, ug/ml)

Whole bacterial antigen	20-I01		20-I04		20-I05		20-I11		20-I15		20-I21		20-I28		20-I35		20-I36	
	**Wk 0**	**Wk 2**	**Wk 0**	**Wk 2**	**Wk 0**	**Wk 2**	**Wk 0**	**Wk 2**	**Wk 0**	**Wk 2**	**Wk 0**	**Wk 2**	**Wk 0**	**Wk 2**	**Wk 0**	**Wk 2**	**Wk 0**	**Wk 2**
**AB005**	<100	<100	242.98	284.84	<100	<100	<100	<100	<100	<100	<100	<100	<100	<100	<100	<100	<100	<100
**AB011**	214.01	212.4	416.84	447.43	189.86	241.38	817.72	1073.1	537.99	426.5	310.96	312.99	295.69	340.48	323.42	336.22	321.29	359.68
**AB015**	<100	<100	122.25	317.04	<100	<100	112.31	4050.9	288.66	3304.9	<100	<100	<100	<100	<100	<100	<100	<100
**AB012**	<100	<100	<100	<100	259.08	138.35	458.93	2804.2	799.48	2234.6	1346.8	1010.3	<100	<100	2897.9	2620.6	<100	<100
**AB021**	<100	<100	162.49	212.4	347.62	342.79	240.01	1393.4	815.69	2096.8	1310.3	943.4	212.51	246.63	2863.8	2686.7	<100	<100
**AB028**	<100	<100	<100	<100	252.64	154.45	442.72	2917.7	767.04	2153.5	1363	915.02	<100	114.39	3023.7	2669.7	<100	<100
**AB004**	<100	100.52	141.56	160.87	769.19	694.36	<100	<100	<100	<100	<100	<100	<100	<100	287.6	282.41	<100	<100
**AB001**	<100	233.29	361.23	409.51	1491	1372.7	45.656	141.87	134.36	134.36	117.82	<100	<100	123.72	721.17	663.08	<100	<100
**AB035**	259.85	416.75	1078.2	1952	1425.8	1411.3	544.78	347.84	607.92	607.92	445.56	412.48	124.75	453.56	1176.5	1075.9	267.89	221.22
**AN006**	<100	<100	327.44	1157.8	105.35	156.05	107.3	155.4	167.43	167.43	<10	<10	<10	111.27	559.36	534.46	<100	<100
**AN023**	539.86	395.03	409.51	1703.4	696.77	479.52	176.45	287.7	186.97	186.97	102.79	<100	<10	<100	<100	106.08	<100	<100
**AN026**	694.36	445.72	346.75	1614.1	691.94	474.69	233.58	320.78	180.96	180.96	<100	<100	<10	<100	<100	<100	<100	<100
**AB ATCC**	<100	<100	<100	<100	<100	<100	<100	<100	<100	<100	<100	<100	<100	<100	<100	<100	<100	<100

Supplementary Figure S4: Subgrouping of clinical *A. baumannii* isolates based on serological recognition by plasma samples from *A. baumannii* infected patients. Paraformaldehyde fixed intact whole cells of bacteria were coated onto plate at 10^6 CFUs. Plasma samples from infected patients at week 0 or week 2 of infection were probed before detected for binding IgG by ELISA.

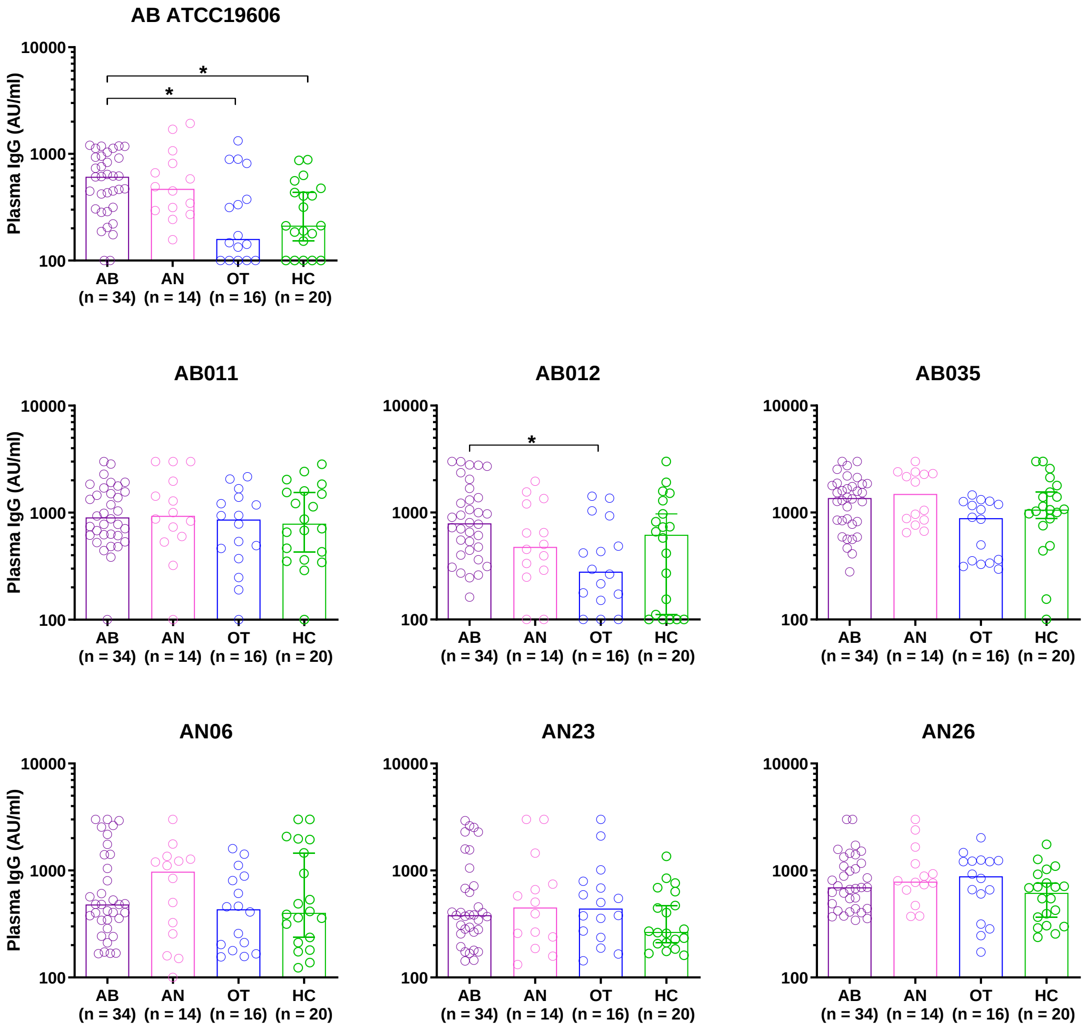

Supplementary Figure S5: Plasma IgG level against intact whole bacterial cells from different type of bacterial infected patients and healthy controls. Paraformaldehyde fixed intact whole cells of *A. baumannii* (AB ATCC, AB011, AB012, and AB035) or *A. nosocomailis* (AN006, AN023, and AN026) bacteria were coated onto plates at 10^6 CFUs. Plasma samples from acute infected patients with *A. baumannii* (n = 34; AB, purple), *A. nosocomailis* (n = 14; AN, pink), other bacteria (n = 16; OT, blue), or healthy controls (n = 20; HC, green) were added before detection of IgG binding by ELISA. Data of plasma IgG in AU/ml was visualized as dot plot with bar at median. Statistically differences were tested using one-way ANOVA with Kruskal-Wallis test, *, $p < 0.05$.
